# Is Repeat PTA of a Failing Hemodialysis Fistula Durable?

**DOI:** 10.1155/2014/369687

**Published:** 2014-01-22

**Authors:** Ioannis Bountouris, Thorarinn Kristmundsson, Nuno Dias, Zbigniew Zdanowski, Martin Malina

**Affiliations:** Skåne University Hospital, Vascular Center, 205 02 Malmö, Sweden

## Abstract

*Purpose*. Our objective was to evaluate the outcome of percutaneous transluminal angioplasty (PTA) and particularly rePTA in a failing arteriovenous fistula (AV-fistula). Are multiple redilations worthwhile? *Patients and Methods*. All 159 stenoses of AV fistulas that were treated with PTA, with or without stenting, during 2008 and 2009, were included. Occluded fistulas that were dilated after successful thrombolysis were also included. Median age was 68 (interquartile range 61.5–78.5) years and 75% were male. *Results*. Seventy-nine (50%) of the primary PTAs required no further reintervention. The primary patency was 61% at 6 months and 42% at 12 months. Eighty (50%) of the stenoses needed at least one reintervention. Primary assisted patency (defined as patency after subsequent reinterventions) was 89% at 6 months and 85% at 12 months. The durability of repeated PTAs was similar to the durability of the primary PTA. However, an early primary PTA carried a higher risk for subsequent reinterventions. Successful dialysis was achieved after 98% of treatments. Nine percent of the stenoses eventually required surgical revision and 13% of the fistulas failed permanently. *Conclusion*. The present study suggests that most failing AV-fistulas can be salvaged endovascularly. Repeated PTA seems similarly durable as the primary PTA.

## 1. Introduction

The number of dialysis patients with end-stage renal failure increases by more than 4% per year in Sweden, with a current prevalence of 900 dialysis patients per million inhabitants [1]. These patients are always concerned about the function of their vascular access because their quality of life depends on it.

Fistulas often stenose and may become dysfunctional. Stenoses necessitate reinterventions or creation of a “de novo” fistula. The number of possible access sites is limited.

**Figure 1:**
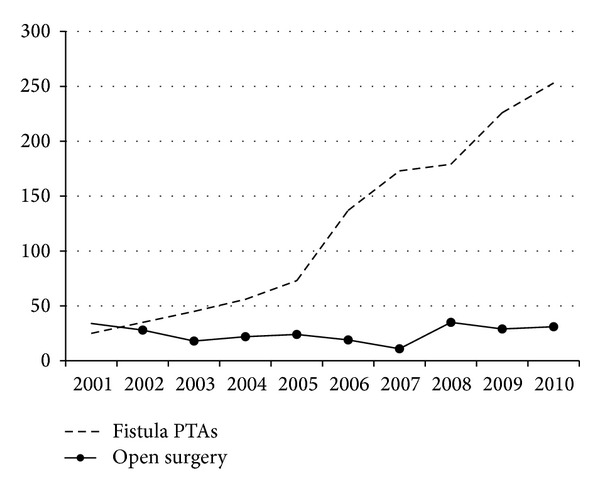
Number of PTAs and surgical operations in hemodialysis fistulas per year at our institution.

PTA has become the treatment of choice for stenoses in AV fistulas, as it is less invasive than open surgery. The reduced need for surgical reinterventions and new fistulas seems to be a direct consequence of this strategy (Figure 1).

The aim of the present study is to assess the durability of PTA in AV fistulas in a center that predominantly uses this technique for salvage of failing fistulas and particularly to evaluate the efficacy of multiple rePTAs: is it worthwhile to dilate a fistula multiple times?

## 2. Patients and Methods

Data from all 106 patients treated with a primary PTA of an AV fistula between January 2008 and December 2009 were prospectively entered into a computerized database. The median age was 68 (interquartile range 61.5–78.5) years, and 75% were male.

All procedures were performed by vascular surgeons or interventional radiologists in a dedicated endovascular suite. Antegrade, retrograde, or both antegrade and retrograde punctures were used, depending on the site of the stenosis as deemed on preoperative ultrasound. A complete angiogram from the proximal arteriovenous anastomosis to the central venous outflow was performed in all cases.

In total, 159 primary stenoses in 106 patients were included in the study. The majority of stenoses, 88%, were in native AV fistulas and the remaining in prosthetic grafts. Baseline clinical characteristics are presented in Table 1.

The majority of the stenoses (51%) were located in radiocephalic fistulas, while 35% were located in brachiocephalic fistulas. The most common site of stenosis in the native AV fistula was the cannulation zone (49%), possibly due to repeat punctures.

Indications for treatment were dysfunction of the fistula with a duplex verified stenosis in 84%, dysfunctional fistula with no duplex examination in 6%, asymptomatic stenosis identified on duplex follow-up in 4%, and occlusion of the fistula in 6%. Nephrologists and dialysis nurses follow up the patients. Duplex ultrasound is routinely performed one month after the creation of a fistula. Thereafter, ultrasonography is only performed in malfunctioning fistulas and in fistulas that are not used for dialysis during a longer period of time.

**Figure 2:**
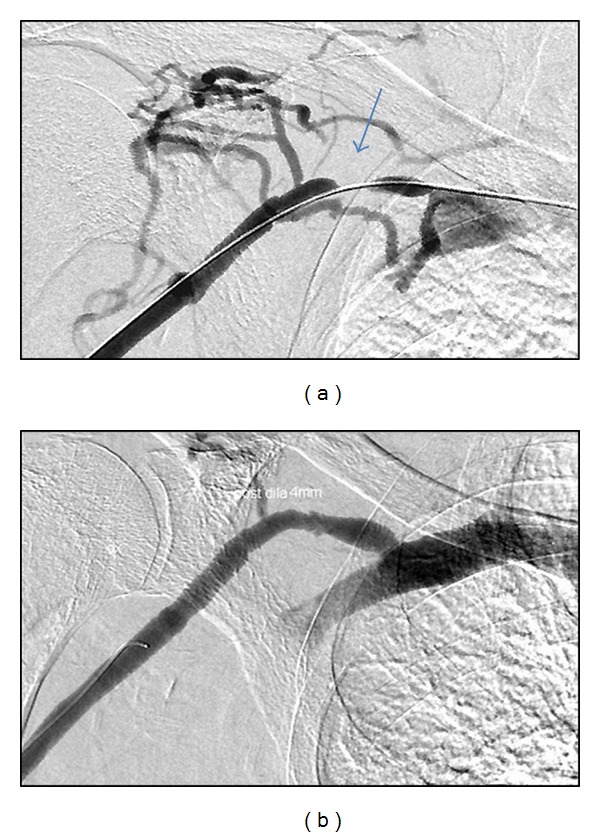
(a) Patient with right radiocephalic fistula and recurrent stenosis of the proximal portion of his cephalic vein (arrow). (b) The lesion has been dilated on three occasions during two years. This is the third PTA. This is particularly important for this patient whose fistulas in the left arm have failed.

Occluded fistulas were thrombolysed with Alteplase (Actilyse, Boehringer Ingelheim) in order to unmask the underlying stenosis which was subsequently dilated. All stenoses were >50% of the lumen diameter. PTA was performed with standard (Figure 2), cutting, or high-pressure balloons as deemed necessary. Balloons 2.5–14 mm in diameter were used (cutting balloons were 3–8 mm). The smallest balloons were used in stenoses that engaged the arterial anastomosis. PTA alone was the primary treatment in 157 cases. Only a covered stent (Fluency-Bard Peripheral, Tempe, Arizona) has been positioned in one patient and a bare stent (Luminex-Bard, Karlsruhe, Germany) has been implanted to another at the primary intervention. Four additional bare stents (2 Protégé-Covidien, Plymouth, Minn, 1 Supera-IDEV, Webster, Tex, 1 Wallstent-Boston Scientific, Natick, MA) and two covered stents (1 Fluency-Bard Peripheral, Tempe, Arizona, 1 Viaban-Gore, Flagstaff, Arizona) have been positioned during reinterventions in other patients. All stents were self-expanding, 6 mm or larger, and dilated at implantation to ensure adequate expansion.

Primary patency was defined as functioning fistula after the first PTA. Primary assisted patency was defined as a functioning fistula after two or more PTAs of that particular segment and secondary patency as a functioning fistula that required an open surgical revision.

The median follow-up was 18.6 (interquartile range 10.4–25.1) months. Twenty-six patients died, but none were lost to follow-up.

## 3. Statistics

Continuous data is presented as median with interquartile range in parenthesis. Reintervention free survival was estimated with life table analysis and illustrated with Kaplan-Meier. One-way ANOVA was used for comparison of time periods between different patient categories. Pearson chi-square was used for comparison of re-interventions in relation to different time periods. All statistical analysis was done in SPSS version 17.0 (SPSS Inc, Chicago, Ill, http://www-01.ibm.com/software/analytics/spss/). A *P* < 0.05 was considered statistically significant.

## 4. Results

Seventy-nine (50%) of the 159 primary PTAs needed no further reintervention.

Eighty (50%) of the primary PTAs required one or more re-interventions. A total of 174 secondary PTAs were carried out during the study period: 36 stenoses (22%) required one reintervention and 44 stenoses (28%) required multiple.

Fourteen (9%) of the 159 stenoses eventually underwent open surgery.

The fistula had to be abandoned due to occlusion or dysfunction during dialysis in 14 (13%) of the 106 patients.

**Figure 3:**
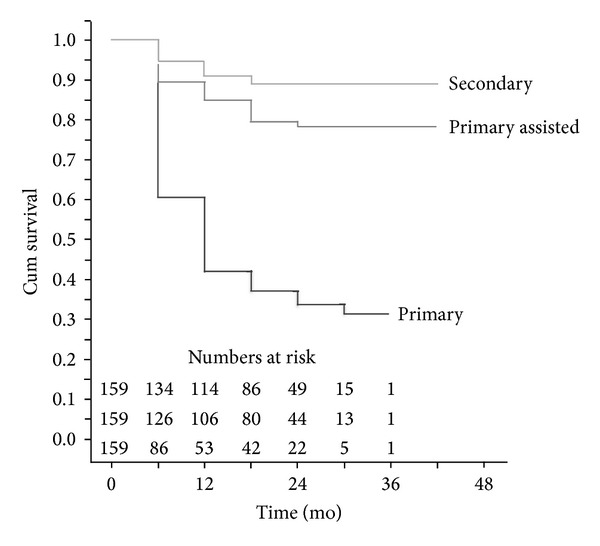
Kaplan Meier estimated patency.

The primary patency of the first PTA was 61 (±4)% and 42 (±4)% at 6 months and 12 months, respectively (Figure 3). The corresponding primary assisted patency was 89 (±3)% and 85 (±3)% while the secondary patency was 95 (±2)% and 91 (±2)%.

**Figure 4:**
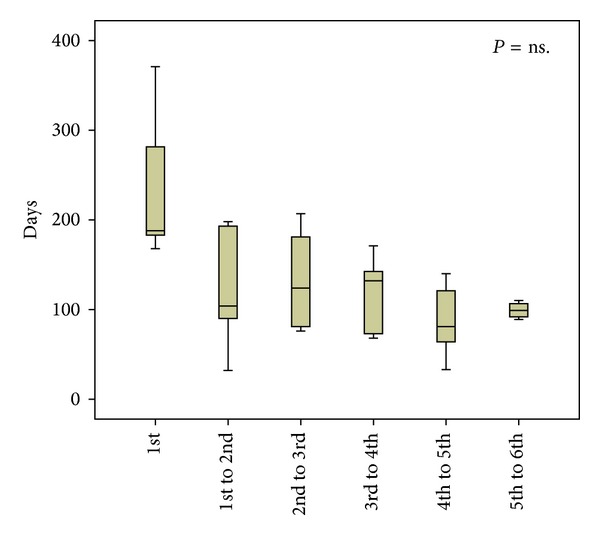
Time intervals between PTAs. Median, IQR in box, and range are given. There was no statistically significant difference between the groups.

The median time from the creation of the fistula to the first PTA was 242 (110–472) days. The time between the first and second PTA was 150 (79.5–265.3) days, between the second and third PTA 106 (82–168) days and between subsequent PTAs 123 (76.5–162), 72 (51–136), and 92 (86.5–102) days, respectively (Figure 4).

**Figure 5:**
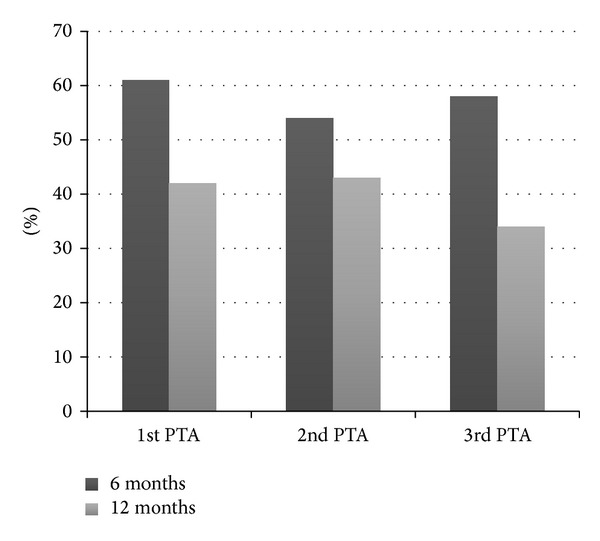
Patency (percentage) without subsequent reintervention after 1st, 2nd, and 3rd PTA, respectively. Black columns represent patency at 6 months and grey columns at 12 months. There is no statistically significant difference in the patency of the 1st, 2nd, and 3rd PTA.

The trend towards a shorter time interval between repeated PTAs was not statistically significant. The durability, that is, patency without subsequent reinterventions, was similar after the 1st, 2nd, and 3rd PTA—both at 6 and 12 months (Figure 5).

There was no correlation between the durability of the first PTA and subsequent ones (*P* = 0.9).

**Figure 6:**
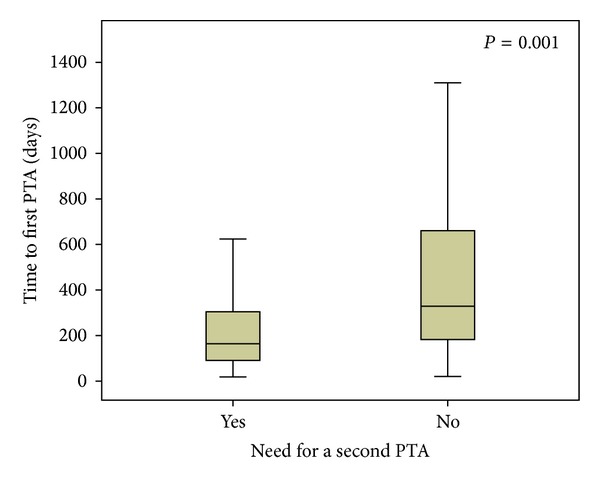
Time of primary PTA in patients who subsequently needed a second reintervention (Yes) and those who did not (No). An early primary PTA is more likely to require a reintervention than a late one.

Stenoses that needed more than one PTA during the study period had the primary PTA significantly sooner than those who did not (164 (90–305) versus 329 (181–674) days, resp., *P* = 0.001) (Figure 6). Patients needing a primary PTA within one year had a threefold risk for additional PTAs (33% versus 11%, *P* = 0.004).

Complications from PTA included minor local hematomas and 5 ruptures (3%). Four ruptures were successfully treated with prolonged balloon inflation and one with external compression.

## 5. Discussion

Patency of AV fistulas can be maintained with both open and endovascular revisions [2–4]. Many investigators feel that the durability of PTA is too short in spite of the fact that results of traditional open surgical patch plasty are scarcely reported [5, 6]. Most papers suggest fewer reinterventions after open surgery but there are no randomised prospective studies comparing open versus endovascular surgery in stenoses of native AV fistulas or prosthetic grafts [7]. According to the National Kidney Foundation (NKF) clinical practice guidelines [8], the choice of open versus endovascular repair should take into account the local expertise of a center. A primary patency of 50% at 6 months seems to be a reasonable goal for endovascular revision.

Endovascular treatment is tempting by offering a rapid procedure with no surgical wound and less need for hospitalization. Furthermore, the fistula can be used for dialysis immediately after PTA while a temporary central dialysis catheter is often required after open repair. For these reasons, PTA is the treatment of choice for failing fistulas at our institution.

Primarily, we used standard PTA balloons that were sized according to the vessel diameter adjacent to the stenosis. High-pressure and cutting balloons were only used if a standard balloon proved insufficient. The same principles are applied by most investigators.

The current study shows that PTA of an AV dialysis fistula offers primary and primary assisted patency that are comparable to or better than most reports from the last decade [9–19]. It is noteworthy that our results were obtained in unselected patients since we offer PTA not only to all patients with a significant stenosis but also to patients with an occluded fistula. The occluded fistulas are dilated after the underlying lesion has been unmasked by thrombolysis or mechanical thrombectomy. Although the primary patency rate may seem low, it implies that half of our patients needed no additional treatment after a single PTA.

Similar reintervention rates to our study were reported by Toya et al. [20]. The question is whether we should insist on spending our resources on endovascular reinterventions or convert to open surgery after failure of the primary PTA. The efficacy of repeat PTA in AV fistulas has been poorly described and restenoses after PTA tend to be regarded as an indication for surgery.

We used open revisions only in specific cases such as pseudoaneurysms and persistent dysfunction of the fistula after PTA. This overall strategy of repeat PTAs with very restrictive use of open revision in nonsalvageable fistulas resulted in a secondary patency rate of 95% and 91% at 6 and 12 months, respectively (Figure 3).

An early first PTA, however, did seem to be associated with a higher risk for additional and more frequent restenoses in that particular segment (Figure 6). This has been previously supported by some authors [21–24] but contradicted by others [25]. It may suggest that different individuals are variously prone to develop venous stenoses. The pathophysiology of restenosis after PTA involves vascular wall injury with potential dissection, injury to the smooth muscle cells, and endothelial layer damage [26–28]. This may trigger cell proliferation [29]. Modification of the vascular wall response by drug eluting balloons or stents may prove beneficial. The data of the present study offer a valuable baseline for such studies.

There were few ruptures from PTA (*n* = 5) and all the ruptures were treated conservatively. Other investigators report ruptures to be potentially more harmful requiring transfusion and stent or stent graft placement [30, 31]. We did not use stents and stent grafts for ruptures. All eight stents and covered stents implanted in this study were used for elastic recoil and only two of them were required at the primary PTA.

Some investigators advocate a prophylactic PTA of stenosis in a well-functioning fistula to improve the survival of the access [32]. Consequently, it supports a surveillance program with duplex ultrasound of AV fistulas [33].

Only 4% of our patients were treated prophylactically based on a duplex ultrasound finding alone and without symptoms of a failing fistula. We achieved a primary assisted patency rate of 85% at one year predominantly treating dysfunctional fistulas.

We therefore believe that regular surveillance and prophylactic PTA are not necessary and that alert clinicians and dialysis nurses are the gold standard for optimal surveillance of AV fistulas. This view is supported by the NKF criteria [8] suggesting that a stenosis >50% should only be treated if the fistula is dysfunctional.

## 6. Conclusions

Failing AV fistulas can be salvaged with PTA. Results fully comparable to open surgery are obtained with less trauma to the patient. RePTA is needed in half the patients but the durability of rePTA is satisfactory and achieves an assisted primary patency rate of 85% at one year. Open surgery should be reserved for nonsalvageable fistulas. Routine surveillance in well-functioning fistulas seems superfluous.

## Figures and Tables

**Table 1:** Baseline clinical characteristics.

Variable	Stenoses, *n* = 159 (%)
Gender	
Male	119 (75%)
Comorbidities	
Coronary artery disease	67 (42%)
Diabetes mellitus	60 (38%)
Type of AV fistula	
Radiocephalic	81 (51%)
Brachiocephalic	56 (35%)
Other native vein	3 (2%)
Prosthetic graft	19 (12%)

Position of stenosis in native AV fistula	*n* = 140 (%)
Anastomosis	40 (29%)
Venous cannulation zone	69 (49%)
Outflow vein	25 (18%)
Central vein	6 (4%)

Position of stenosis in synthetic grafts	*n* = 19 (%)
Arterial anastomosis	3 (16%)
Graft	1 (5%)
Venous anastomosis	11 (58%)
Outflow vein	4 (21%)
Central vein	0

## References

[1] Svenskt Njurregister (SNR) Aktiv uremivård i Sverige1991–2009. http://www.medscinet.net/snr/rapporter.aspx.

[2] Dapunt O, Feurstein M, Rendl KH, Prenner K (1987). Transluminal angioplasty versus conventional operation in the treatment of haemodialysis fistula stenosis: results from a 5-year study. *British Journal of Surgery*.

[3] Hingorani A, Ascher E, Kallakuri S, Greenberg S, Khanimov Y (2001). Impact of reintervention for failing upper-extremity arteriovenous autogenous access for hemodialysis. *Journal of Vascular Surgery*.

[4] Tessitore N, Mansueto G, Lipari G (2006). Endovascular versus surgical preemptive repair of forearm arteriovenous fistula juxta-anastomotic stenosis: analysis of data collected prospectively from 1999 to 2004. *Clinical Journal of the American Society of Nephrology*.

[5] Ljungström KG, Troeng T, Björck M (2008). Time-trends in vascular access surgery in Sweden. *European Journal of Vascular & Endovascular Surgery*.

[6] Napoli M, Prudenzano R, Russo F, Antonaci AL, Aprile M, Buongiorno E (2010). Juxta-anastomotic stenosis of native arteriovenous fistulas: surgical treatment versus percutaneous transluminal angioplasty. *The Journal of Vascular Access*.

[7] Campos RP, Do Nascimento MM, Chula DC, Do Nascimento DE, Riella MC (2006). Stenosis in hemodialysis arteriovenous fistula: evaluation and treatment. *Hemodialysis International*.

[8] NKF-DOQI clinical practice guidelines for vascular access (1997). Dialysis outcomes quality initiative. *American Journal of Kidney Diseases*.

[9] Turmel-Rodrigues L, Pengloan J, Bourquelot P (2002). Interventional radiology in hemodialysis fistulae and grafts: a multidisciplinary approach. *CardioVascular and Interventional Radiology*.

[10] Šurlan M, Popovič P (2003). The role of interventional radiology in management of patients with end-stage renal disease. *European Journal of Radiology*.

[11] Haage P, Günther RW (2006). Radiological intervention to maintain vascular access. *European Journal of Vascular and Endovascular Surgery*.

[12] Miquelin DG, Reis LF, da Silva AA (2008). Percutaneous transluminal angioplasty in the treatment of stenosis of arteriovenous fistulae for hemodialysis. *International Archives of Medicine*.

[13] Greenberg JI, Suliman A, Angle N (2008). Endovascular Dialysis Interventions in the Era of DOQI. *Annals of Vascular Surgery*.

[14] Flu H, Breslau PJ, Straaten JMK-V, Hamming JF, Lardenoye J-WH (2008). The effect of implementation of an optimized care protocol on the outcome of arteriovenous hemodialysis access surgery. *Journal of Vascular Surgery*.

[15] Hansen MA, Gibsholm-Madsen K, Christensen T (2009). Endovascular treatment of dysfunctional haemodialysis fistulas. *Ugeskr Laeger*.

[16] Cohen A, Korzets A, Neyman H (2009). Endovascular interventions of juxtaanastomotic stenoses and thromboses of hemodialysis arteriovenous fistulas. *Journal of Vascular and Interventional Radiology*.

[17] Patanè D, Morale W, Malfa P (2009). Steno-obstructions of haemodialytic FAV: new aspects of endovascular treatments. *Giornale Italiano di Nefrologia*.

[18] Kim WS, Pyun WB, Kang BC (2011). The primary patency of percutaneous transluminal angioplasty in hemodialysis patients with vascular access failure. *Korean Circulation Journal*.

[19] Ayez N, Fioole B, Aarts RA (2011). Secondary interventions in patients with autologous arteriovenous fistulas strongly improve patency rates. *Journal of Vascular Surgery*.

[20] Toya N, Fujita T, Hagiwara H (2006). Periodic duplex ultrasonography screening together with elective percutaneous transluminal angioplasty in the management of graft arteriovenous fistulas for hemodialysis. *Surgery Today*.

[21] Turmel-Rodrigues L, Pengloan J, Baudin S (2000). Treatment of stenosis and thrombosis in haemodialysis fistulas and grafts by interventional radiology. *Nephrology Dialysis Transplantation*.

[22] Turmel-Rodrigues L, Mouton A, Birmelé B (2001). Salvage of immature forearm fistulas for haemodialysis by interventional radiology. *Nephrology Dialysis Transplantation*.

[23] Heye S, Maleux G, Vaninbroukx J, Claes K, Kuypers D, Oyen R (2012). Factors influencing technical success and outcome of percutaneous balloon angioplasty in de novo native hemodialysis arteriovenous fistulas. *European Journal of Radiology*.

[24] Caeiro F, Carvalho D, Cruz J (2013). Efficacy of percutaneous transluminal angioplasty on dysfunctional fistulae because of inflow stenosis. *The Journal of Vascular Access*.

[25] Manninen HI, Kaukanen ET, Ikäheimo R (2001). Brachial arterial access: endovascular treatment of failing Brescia-Cimino hemodialysis fistulas—initial success and long-term results. *Radiology*.

[26] Davidson CJ, Newman GE, Sheikh KH, Kisslo K, Stack RS, Schwab SJ (1991). Mechanisms of angioplasty in hemodialysis fistula stenoses evaluated by intravascular ultrasound. *Kidney International*.

[27] Higuchi T, Okuda N, Aoki K (2001). Intravascular ultrasound imaging before and after angioplasty for stenosis of arteriovenous fistulae in haemodialysis patients. *Nephrology Dialysis Transplantation*.

[28] Roy-Chaudhury P, Sukhatme VP, Cheung AK (2006). Hemodialysis vascular access dysfunction: a cellular and molecular viewpoint. *Journal of the American Society of Nephrology*.

[29] Chang C-J, Ko P-J, Hsu L-A (2004). Highly increased cell proliferation activity in the restenotic hemodialysis vascular access after percutaneous transluminal angioplasty: implication in prevention of restenosis. *American Journal of Kidney Diseases*.

[30] Raynaud AC, Angel CY, Sapoval MR, Beyssen B, Pagny J-Y, Auguste M (1998). Treatment of hemodialysis access rupture during PTA with Wallstent implantation. *Journal of Vascular and Interventional Radiology*.

[31] Bittl JA (2009). Venous rupture during percutaneous treatment of hemodialysis fistulas and grafts. *Catheterization and Cardiovascular Interventions*.

[32] Tessitore N, Mansueto G, Bedogna V (2003). A prospective controlled trial on effect of percutaneous transluminal angioplasty on functioning arteriovenous fistulae survival. *Journal of the American Society of Nephrology*.

[33] Scaffaro LA, Bettio JA, Cavazzola SA (2009). Maintenance of hemodialysis arteriovenous fistulas by an interventional strategy: clinical and duplex ultrasonographic surveillance followed by transluminal angioplasty. *Journal of Ultrasound in Medicine*.

